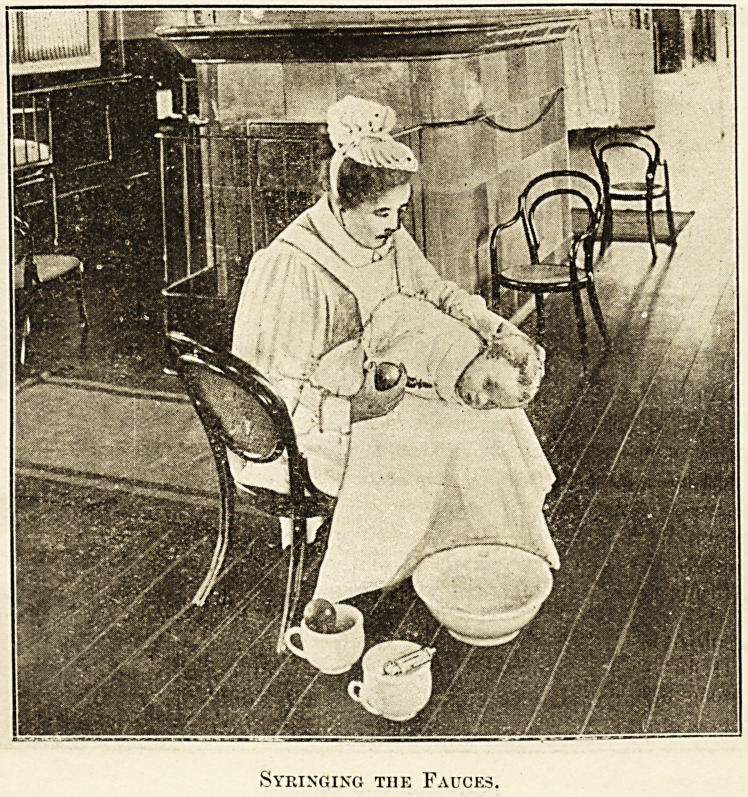# On the Methods of Isolation and the Present Treatment of Infectious Diseases

**Published:** 1899-04-15

**Authors:** R. A. Birdwood

**Affiliations:** Medical Superintendent, Park Hospital


### Hospital Clinics and Medical Progress.

### ON THE METHODS OF ISOLATION AND THE PRESENT TREATMENT OF INFECTIOUS DISEASES.

By R. A. Birdwood, M.A., M.D., Medical Superintendent, Park Hospital.

In the hospitals of the Metropolitan Asylums Board accommodation is provided for London patients suffering from some of the infectious diseases. The Board's work began in 1870 with the admission of relapsing fever and small-pox. In the following year typhus, enteric, and scarlet fevers were admitted. In 1888 diphtheria was added to the list. In that year only one patient suffering from typhus was admitted, whilst in the following year five small-pox patients formed the total admissions for that disease.

A Ward in a Fever Hospital.

The Metropolitan District extends for ten miles from Hampstead Heath to the Crystal Palace, and for fifteen miles from Bostall Heath to Wormwood Scrubs. The estimated population resident within the boundaries being about four-and-a-half millions. For this population more than six thousand beds are provided for fever and diphtheria patients in hospitals at Homerton, Tottenham, Hampstead, and Fulham on the north of the Thames; at Tooting, Stockwell, Deptford, Hither Green, and Shooter's Hill on the south of the river. There is a hospital for convalescents at Winchmore Hill, and another is about to be built at Carshalton. Small-pox patients are taken down the river in ambulance steamers to the hospital ships off Purfleet. The hospital for small-pox convalescents is at Darenth. A new hospital is being built near the river to replace the ships for acutely ill patients.

If a person suffering from one of the diseases admitted, or the relatives responsible for the care of the patients, wish to have the patient removed from home to be treated in hospital, all that is necessary to be done is for a registered medical practitioner to certify that the patient has small-pox, diphtheria, scarlet, enteric or typhus fever. The substance of this certificate with the patient's age and address should be communicated in the most expeditious way to the Clerk to the Board by day, or to the superintendent of the nearest ambulance station by night. If a bed is available, an ambulance with a nurse will arrive shortly afterwards. The ambulances are admirably constructed and are provided with an air mattress, hot-water bottles and such light refreshments and restoratives as are likely to be required on the journey.

The managers of the Asylums Board are responsible for the patient only. The disinfection of the dwelling and clothing is carried out by the servants of another authority (vestries, &c.), whilst in the event of small-pox, if it is deemed advisable to vaccinate such other inmates as have been exposed to infection, the duty is entrusted to a third public body (the Guardians of the poor).

The removal of the infectious patient from home to hospital constitutes the first stage of the process of isolation designed for the prevention of the spread of disease to the community. The disinfection of the dwelling and clothing; the examination, and, if necessary, rectification of any insanitary state at home, is the next stage. In the case of small-pox, revaccination of all persons exposed to infection should be advised, unless they are protected by a previous attack of small-pox or otherwise. Lastly, the patient is detained in hospital till considered free from the risk of infecting others, whilst the staff in attendance on the sick adopt certain obvious precautions before going out of the hospital.

The Park Hospital at Hither Green is one of the recently structed of these hospitals. It covers about twenty acres, and has less than thirty beds to an acre. No part of any ward is within one hundred feet of the boundary. A group of pavilions for 368 scarlet fever patients is on one side of the hospital; another group for 120 diphtheria and enteric fever patients is on the other. Behind these wards are a number of small wards of four beds each, and others of one bed each for the isolation of outbreaks of measles, chicken-pox, or whooping cough amongst the other patients. Altogether 60 beds are provided for this purpose. The diphtheria and enteric fever wards have fifteen feet of wall space allowed for each bed, whilst in the wards for scarlet fever patients twelve feet suffice. All the staff brought into contact with the patients or infected articles (including medical officers, nurses, laundry maids, and porters) reside within the hospital bounds. None of the uniform worn in hospital is worn outside. Even every letter sent from the wards is disinfected by steam in a Lyon's machine.

The Distribution of the Metropolitan Fever Hospitals.

N. Norfolk House, Norfolk Street, Strand.

1. North-Eastern Hospital, Tottenham.

2. North-Western Hospital, Hampstead.

3. Eastern Hospital, Homerton.

4. Western Hospital, Fulham.

5. Grove Hospital, Tooting.

6. Fountain Hospital, Tooting.

7. South-Western Hospital, Stockwell.

8. South-Eastern Hospital, New Cross.

9. Brook Hospital, Shooter's Hill.

10. Park Hospital, Hither Green.

A. West Wharf, Fulham.

B. South Wharf, Rotherhithe.

C. North Wharf, Blackwall.

Passing from the methods of isolation to the routine treatment of scarlet fever and diphtheria patients in this hospital it is perhaps well to point out at first that there is no specialism in fever practice. We may have as complications brain, lung, heart, or abdominal disease; or an epidemic may occur in the wards of a children's or general hospital, and any of the patients from that hospital may be transferred to one of the fever hospitals. There are, however, two facts that make a difference: the one is that the vast majority of scarlet fever and diphtheria patients are children, and the other that from the large number of similar conditions dealt with the nurses become expert at various toilet and other manipulations. The child's comfort and welfare are dependent on the skill with which these proceedings are carried out.

In an ordinary mild, uncomplicated attack of scarlet fever the child is kept in bed for three weeks and given low diet, with stewed fruit, till three days after the temperature has fallen to normal. The bowels are opened daily, and the urine tested for albumen on alternate days. The reason for so long a stay in bed is that if nephritis supervenes it usually does so about the end of the third week, and the maintenance of the regular action of the bowels seems to have a marked and beneficial effect in preventing œdematous swellings of the legs. They are also detained in bed whilst albuminuria or dilated heart persists, and when in bed are blanket-bathed daily. It is not prudent to use the bath during this time, as faintness or a bad colour come on occasionally. If all goes well a warm soap-and-water bath is taken three times a week during convalescence. As a rule, this is sufficient treatment for desquamation. If at the end of six weeks from the onset of illness all symptoms have ceased the patients are discharged, being given a final bath and dressed in their own clothes after leaving the ward. If desquamation continues, a weak acid solution, such as an ounce of dilute acetic acid to half a pint of water, applied to the soles or palms on lint for a quarter of an hour, or else rubbing with glycerine and borax, does much to remove adherent epithelium. Desquamation may go on for three or four months or even longer, and it is quite common for it to come on twice and sometimes three times. It is difficult to believe that these recurrent desquamations are infectious.

The high temperature of scarlet fever is well controlled by sponging with tepid water, and the patient generally feels better after it is done. It is good practice to sponge whenever the four-hourly temperature exceeds 39 deg. C. Hyperpyrexia of scarlet fever is not controlled by baths nor drugs; the former may be frequently repeated, but the temperature rises again and the patient becomes rapidly worse. Now and again a patient recovers after a temperature of 41 deg. C., and this does encourage one to keep on with tepid sponging. An undoubtedly good effect is sometimes observed in the reduction of temperature on removal of blankets and leaving the patient covered with a sheet only. If either sponging or removal of blankets induces shivering or a feeling of coldness, or actual coldness of the extremities, it should be discontinued and warm water bottles should be used. A thirsty fever patient should be given plenty of drink, and it is well to remember that there is sometimes a repugnance to milk. Water relieves thirst best and should be given. Grape or orange juice is generally liked and does good. Barley water with lemons, or soda water, or lemonade are suitable drinks. For the sore throat we use a chlorine gargle (chlorate of potash ℥iss., hydrychloric acid ʒvj. with five pints of water added after the evolution of the gas) when an acid preparation is desired; or Liq. sodæ chlorinatæ 1 in 15 of water when an alkaline. Equal parts of either of these chlorine solutions and warm water are mixed just before use. If the patient is not old enough to gargle, a ball syringe with a long nozzle attached is used for flushing out the fauces. Two 4 oz. syringefuls are enough. Should the patient resist the attempt to pass the nozzle between the teeth, there is no occasion to use force to do so. The fauces can be well washed by passing the nozzle between the cheek and the teeth so that its point goes just beyond the last molar tooth. This should be done first on one side, then on the other. The patient should be held with the face downwards. The practice of gargling or syringing the throat is far better than swabbing; for the tissues are often soft enough to be damaged by the latter proceeding.

If swabbing is ordered the finger should not be used for this purpose. First, because of the risk of conveying the infection of post scarlatinal diphtheria from one scarlet fever patient to another; as in practice the fingers cannot be sterilised between the swabbing of successive patients. Secondly, because nurses who carry out swabbing of the fauces sooner or later have an obstinate ulceration at the roots of the nails; and

Swabbing the Throat.

lastly, they are liable to painful bites. The proper method is for the patient to be wrapped round and placed on the back at the edge of the cot, with the face inclined to the window so as to illuminate the back of the throat. The nurse should stand between the window and the patient; the assistant on the other side of the cot. The latter should hold the patient's head and body steady. The former should pass a bone spatula between the teeth with her left hand and swab with the holder in the right, taking care to see what she is doing. The throat is hardly ever so painful as to require cocaine. If it is used care is required in feeding the patient till the effect of the drug has passed away.

For discharges from the nose and ear a solution of boric acid (℥ij.to five pints) is used; before use it is mixed with an equal quantity of warm water. A two-ounce ball syringe with a blunt pointed nozzle is best for the nose, and a one-ounce glass syringe with a projecting ridge on the nozzle for the ear so as to prevent injury to the internal parts. Accurate plugging of the nostrils with cotton wool often succeeds in stopping a troublesome and profuse discharge from the nose. The way to carry out these washings of the fauces, nose, and ears differs for babies and older children. If the patient is a baby, after applying a wrap to prevent struggling, the nurse should sit down on a low chair, place a towel over her skirt, then place the baby on her lap with the head to the right and the face downwards but turned a little toward her, so as to enable her to see what she is doing. The nurse's left forearm should steady the baby's body and the left hand should firmly hold the back of the child's head. A basin, lotions, syringes should be arranged on the floor within reach. The mouth should be washed out first, then the nose, and after that the right ear. The baby is then turned over and has the left ear syringed. This can all be done without an assistant. When a nurse becomes expert at it, it is the method that causes the least discomfort to the patient, and at the same time is the most efficient toilet of the parts. With an older child the same routine is gone through with the patient at the edge of the cot and turned well over, so that whatever fluid is used falls out. An assistant is required, especially if the child resists. All nozzles, swab holders, and spatulas should be boiled before use for each patient. The syringes should be rinsed with water immediately they are emptied as the chlorine and acid injures them.

For nephritis we usually order loin poultices or fomentations. In some instances the drawing of a few ounces of blood has been followed by a flow of urine; in others no such result has ensued. Seidlitz powders or compound liquorice powder should be given, not calomel. Whenever a patient has any difficulty in swallowing, or when coughing is induced on the attempt to drink, nasal feeding should be resorted to. Milk and water are administered by a catheter passed through the nose. An extra length of tubing is attached to a flexible catheter and a glass syringe with the piston removed is fixed to the other end of the tube. The food should never be poured into the glass receiver till it is certain that the catheter is not in the trachea. Enlarged tonsils, adenoids or post-pharyngeal abscess cause an obstruction at times in the feeding of the patient by this means, but, as a rule, there is no difficulty, and it may even be carried out without awakening a sleeping baby. When all the liquid has passed, the catheter should be tightly pressed between the finger and thumb and quickly withdrawn. The catheter is boiled before use.

The rheumatism of scarlet fever yields readily to salicylate of soda, and is rarely followed by endocarditis or chorea. Pus in the joints is an occasional sequel of septic throats. The joints should be opened early. The most common conditions requiring operative interference are abscesses and broken-down glands in the neck, post-pharyngeal abscess, mastoid disease following otorrhœa, empyema, and enlarged tonsils. In the treatment of scarlet fever and diphtheria at this hospital, as a rule, no alcohol is given to the patient. In the rare instances when it is prescribed very small doses are given.

In the local throat treatment of diphtheria the same practice is followed as for scarlet fever. Antitoxin is given in the following doses: For ordinary mild cases, 2,000 units on the first day; for severe cases, 8,000 to 12,000 units when the patient is first seen, followed by 2,000 to 8,000 every 12 hours for the next 24 or 48 hours, according to the gravity of the case and the persistence of the local exudation. In the result this treatment is frequently disappointing. Roux's syringe is used, and the injection is made in the loin after washing. A little blue gauze with collodium is pressed over the puncture, and kept in position with strapping. For cardiac irregularities and paralysis the patients are kept in bed, the sub-normal temperatures of diphtheria being also an indication for rest. When the vomiting of vagus paralysis comes on rectal feeding and enemata of warm water are employed. For paralysis of the diaphragm the foot of the bed is raised a foot or more to assist in the expulsion of phlegm. The adoption of this method seems to be of great advantage to the patient.

Syringing the Fauces.

For obstruction of breathing tracheotomy is usually performed at this hospital. Intubation is but rarely done. The operation is performed early; with two exceptions. We delay doing tracheotomy for a short time when the distress has come on in the ambulance, hoping that the exposure to cold on the journey may have induced an œdematous obstruction, and that this may pass off with the improved environment. If the obstruction begins about the fifth day of the illness, it may be caused by membrane beginning to come away. It is hoped here that with the expulsion of membrane the need for opening the trachea may end. It is, however, only allowable to delay if a medical man is at hand. The bronchitis kettle and artificial fog are not employed. To its abandonment some of the reduced mortality after operation is due.

I have attempted to briefly indicate the work of a fever hospital within the title limitation of this paper. The treatment of disease must be learnt at the bedside, and that of fevers perhaps more than that of any other class of disorders. Every year, however, sees an increasing proportion of the cases of infectious disease removed to isolation hospitals, and it is at these institutions that the clinical teaching of the treatment of fevers must be undertaken. As far as the medical profession is concerned every student attends a fever hospital. I often wonder when trained nurses will think it necessary to do so. My advice to them is that if after their training they have a chance of nursing in a fever hospital for six months or more, not to neglect the opportunity. It will be a most valuable experience.

I am indebted to Mr. J. W. Nevill for the illustrations.

## Figures and Tables

**Figure f1:**
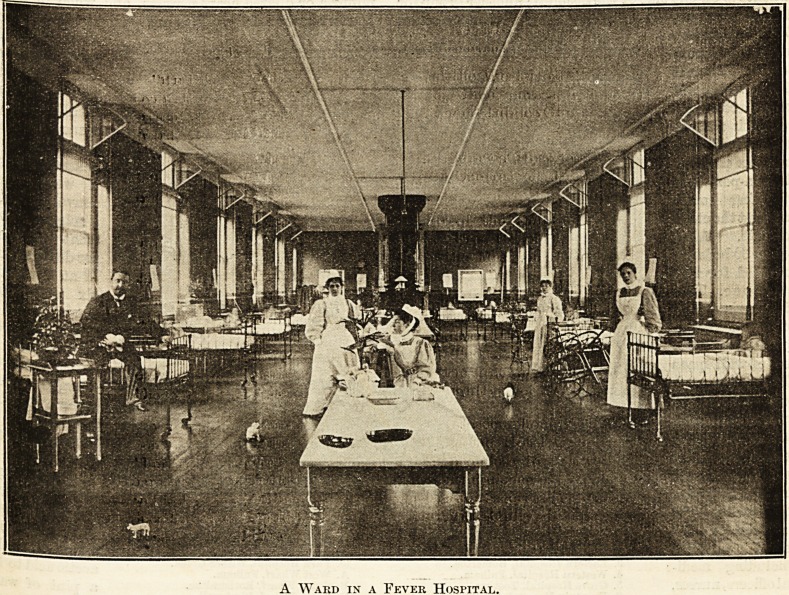


**Figure f2:**
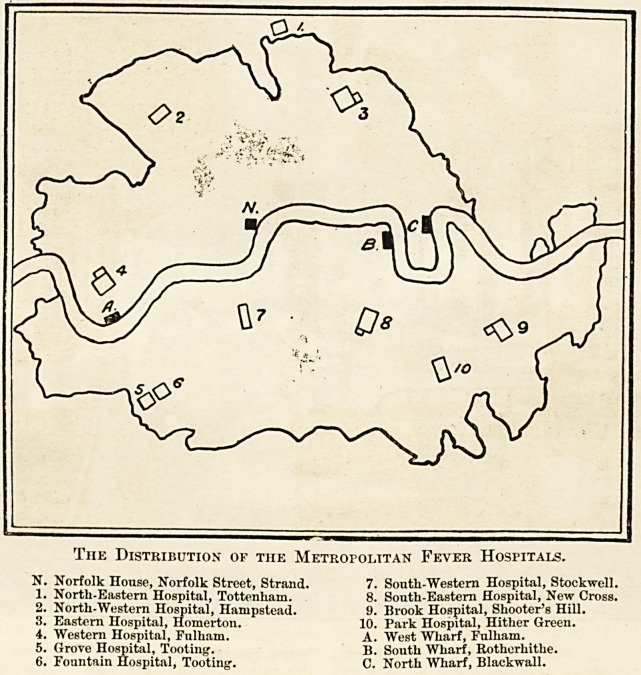


**Figure f3:**
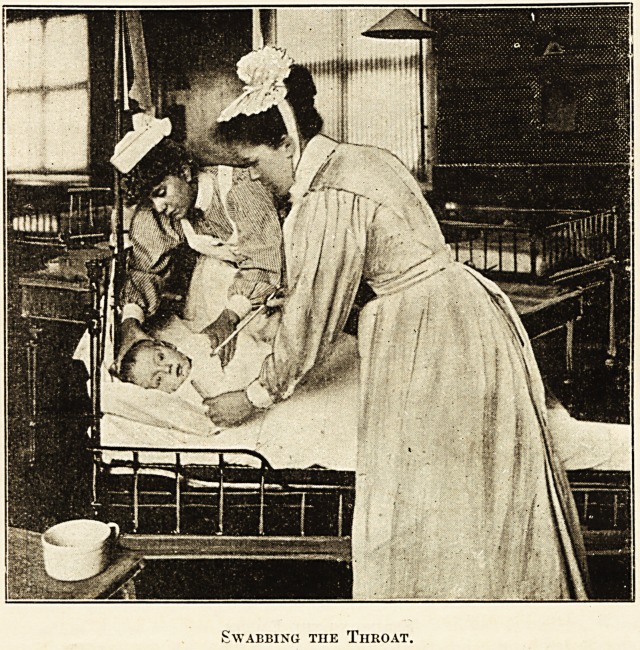


**Figure f4:**